# Assessing the Roles of Aging, Estrogen, Nutrition, and Neuroinflammation in Women and Their Involvement in Alzheimer’s Disease—A Narrative Overview

**DOI:** 10.3390/ijms27031239

**Published:** 2026-01-26

**Authors:** Edwin D. Lephart, K. Scott Weber, Dawson W. Hedges

**Affiliations:** 1 Department of Cell Biology & Physiology, The Neuroscience Center, College of Life Sciences, Brigham Young University, Provo, UT 84602, USA; 2 Department of Microbiology and Molecular Biology, College of Life Science, Brigham Young University, Provo, UT 84602, USA; scott_weber@byu.edu; 3 Department of Psychology (Cognitive Behavior), The Neuroscience Center, College of Family, Home and Social Sciences, Brigham Young University, Provo, UT 84602, USA; dawson_hedges@byu.edu

**Keywords:** cognitive function, Alzheimer’s disease, estrogen, perimenopause, menopause, aging, nutrition

## Abstract

The purpose of this narrative review is to examine women’s cognitive health and to highlight its association with four major pillars: (1) aging, (2) estrogen decline and loss, (3) diet, and (4) neuroinflammation, and their contribution to cognitive decline, with a focus on this combination to increase awareness and address the progression and potential amelioration of Alzheimer’s disease (AD). Often overlooked, estrogen decline during perimenopause and loss of estrogen production from the ovaries after menopause negatively influences almost every tissue and organ in the body, including the brain. This estrogen loss leads to inflammation, as can poor nutritional choices, both of which have a profound impact on short- and long-term health and can increase the risk of dementia, including AD. Thus, this overview covers the following four pillars (1) a brief background on cognitive decline and AD with aging, (2) the importance of and changes in estrogen with aging, (3) influence of dietary choices on overall well-being and brain health, and (4) the biochemical and molecular mechanisms by which this combination of factors may lead to neuroinflammation, resulting in cognitive decline and AD. Finally, this review briefly presents a hypothesis on whether women during perimenopause should be administered estrogen to span the transition into menopause to protect against cognitive decline and possibly ameliorate the risk of AD. This article is based on previously conducted studies and does not contain new data/results (studies) of human participants or animals performed by the authors.

## 1. Introduction

Someone in the world develops dementia every 3 s [1,2]. There were over 55 million people worldwide living with dementia in 2020, a number projected to increase to 153 million by 2050 [2]. The annual global cost of dementia is above in United States Dollars (USD) 1.3 trillion and is expected to rise to USD 2.8 trillion by 2030 [1,2]. The female-based prevalence of Alzheimer’s disease (AD) is well documented, with postmenopausal women accounting for over sixty percent of all those affected, which is only partially explained by survival rates and longevity [3]. In this regard, women have a higher incidence of AD partly because they live longer, since increasing age is the biggest risk factor for AD; however, it is not the only reason. Research points to biology, including estrogen decline and loss at menopause, genetics, and social factors that contribute to the higher prevalence of AD in women [3,4]. In addition, women have a higher incidence and prevalence of AD and show faster cognitive decline and pathological burden after diagnosis [5,6]. Thus, women represent a critical demographic for the prevention and delay of the onset of AD [3,4].

The purpose of this narrative overview is to evaluate the vital roles of aging and estrogen as essential functions in women’s health and to highlight other significant pillars, such as diet and neuroinflammation, and their contribution to cognitive decline, with a focus on this combination to increase awareness and address the progression and potential amelioration of AD. For example, the latest Lancet Commission report on dementia provides evidence about dementia prevention, intervention, and care, and several potentially modifiable risk factors for AD have been proposed (such as low educational attainment, hearing loss, hypertension, smoking, obesity, depression, physical inactivity, diabetes, excessive alcohol consumption, traumatic brain injury, air pollution, and social isolation) [7]. However, there is a need to address potentially modifiable risk factors for dementia in aging women. Often overlooked is the fact that estrogen decline during perimenopause and loss of estrogen production from the ovaries after menopause can negatively influence almost every tissue and organ in the body [8,9]. This estrogen decline can lead to inflammation and neuroinflammation, which increases AD risk. Both inflammation and poor nutritional choices can have a profound impact on short- and long-term health and can increase the risk of dementia and AD in women. Thus, this overview covers (1) a brief background on cognitive decline and AD with aging, especially in women, (2) the importance of and changes in estrogen with aging, (3) how dietary choices influence overall well-being and brain health, and (4) the biochemical and molecular mechanisms by which this combination of factors can lead to neuroinflammation, cognitive decline, and AD. Finally, this overview presents the hypothesis of whether women during perimenopause should be administered estrogen to span the transition into menopause and provide protection against cognitive decline and possibly ameliorate or delay the onset of AD.

This narrative overview used foundational figures, graphics, and tables that depict recent literature results covering the topics/pillars listed above, mainly from the last five years (from January 2020 to November 2025), with general and more recent reports included where applicable. These topics were explored using the following keywords: cognitive decline, Alzheimer’s disease, estrogen, aging, diet, nutrition, inflammation, and estrogen receptors, using the following databases: PubMed, Science Direct, Scopus, and Google Scholar. Some references were included without a year interval range limit, which provided data and background information on various topics such as biochemical and molecular mechanisms for the factors searched. We addressed the limitations of selection and interpretation bias by defining the topics/pillars, concepts, and principles investigated and by capturing the most up-to-date research available to present a clear understanding of the themes and hypotheses covered in this narrative overview. In general, references focused mainly on human investigations that included observational studies, comprehensive reviews, systematic, and meta-analyses where accessible and appropriate.

## 2. Aging: Cognitive Decline and Alzheimer’s Disease

### 2.1. Background

The first pillar presented is aging, where AD represents approximately 70 percent of all dementias [1,2]. As populations around the world continue to age, the challenges associated with growing older have become an increasingly urgent public health concern [7]. Among these challenges, AD stands out as one of the most profound and devastating conditions affecting older adults [7]. Aging is the single greatest risk factor for Alzheimer’s disease, as the gradual biological changes that occur over time can impair brain function and increase vulnerability to neurodegeneration [7,8]. Understanding Alzheimer’s disease through the lens of aging not only highlights why its prevalence rises sharply in later life but also underscores the importance of addressing age-related processes in efforts to prevent, manage, and ultimately treat this progressive disorder [7].

Therefore, aging is a crucial risk factor for AD, a progressive brain disorder that causes memory loss, confusion, and difficulty with daily tasks [7,8,9]. Symptoms increase over time, leading to severe cognitive decline, behavioral changes like mood swings, and loss of physical functions, like swallowing and walking [7,8,9]. While some cognitive functions slow as a normal aspect of aging, AD involves significant and irreversible damage to brain cells via various intracellular and extracellular alterations [7,8,9].

### 2.2. Aging, Cognitive Decline and Factors Associated with AD

Although numerous factors, including low educational attainment, tobacco smoking, alcohol use, decreased hearing acuity, hypertension, and diabetes, have been associated with the incidence of AD [7,8,9], age is the strongest risk factor for AD and related dementias [6,7,8]. Even apart from neurodegenerative disease, cognitive function tends to decrease across the lifespan beginning in early adulthood [7,9,10,11,12]. For example, neural processing speed can start to decline after peaking around age 18 to 19 years, while some studies suggest that other age-related cognitive decline begins in healthy adults in their 20s and 30s [11,12]. Short-term memory can begin to decline around age 35 years, but the most noticeable changes in slower processing speed may become apparent at age 60 years and older [11,12].

Susceptibility to cognitive decline and AD is influenced not only by age but also by sex, with women representing nearly two-thirds of people in the United States (USA) with AD, and a woman’s estimated lifetime risk of developing the disease at age 65 is one in six, compared to one in eleven for a man [8,13]. However, the reasons for this sex difference remain unclear [13]. Furthermore, aging is associated with poorer quality of sleep and more sleep pathology, factors that themselves appear strongly related to mild cognitive impairment and dementia [14,15]. Numerous age-related neuroanatomical, neurophysiological, metabolic, epigenetic, and biochemical changes likely related to AD and other dementias can occur with aging, such as changes in synaptic, mitochondrial, immunological function, and gray and white matter volumes [14,15,16,17]. 

The core clinical characteristics include progressive memory loss (especially short-term memory), impaired learning and recall, disorientation (in time and place and later to people), language difficulties (reduced fluency and difficulty finding words to express), impaired executive function (planning, problem solving, judgment), and visuospatial deficits (getting lost and recognizing objects and faces) [1,2,8].

Behavioral and psychological symptoms include personality and mood changes, depression, agitation/irritability, delusions/hallucinations, and sleep disorders [1,2,8].

In addition to these changes, increased amyloid-beta, hyperphosphorylated tau protein, cellular senescence, and inflammation increase with both age and AD [9,10,15]. For example, extracellular deposits of beta-amyloid protein disrupt communication between neurons [9,10,15] and neurofibrillary tangles, which are intracellular aggregates of hyperphosphorylated tau protein, can cause collapse of neuronal transport systems [9,10,15]. Both of these neuropathological processes lead to neuroinflammation that causes progressive loss of neurons and synapses along with marked atrophy of the hippocampus and cerebral cortex and enlargement of the brain ventricles [9,10,15]. Finally, increasing oxidative stress is associated with aging and AD [9,15,16,17]. While the focus of this presentation is on AD, it is important to distinguish this disorder from other dementias; therefore, Table 1 displays a comparison between Alzheimer’s disease versus other dementia characteristics (see below) [1,2,3,7,10,11,13].

Despite the numerous factors associated with the risk of cognitive decline and AD, the increased risk of AD in women merits further investigation into the possible role of age-related decreasing concentrations in estrogen on the risk of AD [6,9,14,18].

## 3. The Importance of and Changes in Estrogen with Aging

As women age, physiological changes occur across multiple systems, many of which are driven by shifts in hormonal regulation. Among these, alterations in estrogen levels play a central role in shaping the aging process [19]. Estrogen, a key hormone involved in reproductive, bone, cardiovascular health, and metabolic balance, declines with age, particularly during the menopausal transition [19]. This hormonal change not only marks a significant milestone in women’s reproductive aging but also contributes to a range of systemic effects that influence health outcomes later in life [19,20]. Understanding how aging intersects with changes in estrogen provides critical insight into the biological mechanisms underlying age-related conditions in women [21]. Therefore, the second pillar presented herein is the importance of and changes in estrogen with aging in relation to AD [21].

### 3.1. Estrogen Is Vital for Women’s Health

Estrogen is essential to female health [19,20,21]. For example, estrogen plays key roles in physiological and reproductive functions, maintenance of bone mineral density, cardiovascular regulation, central nervous system and cognitive processes, integumentary health, and the modulation of mood and sleep–wake cycle [19,20,21]. A decline in estrogen levels, such as occurs during perimenopause and especially at menopause, can lead to hot flashes, night sweats, palpitations, mood changes and anxiety, and an increased risk of conditions like osteoporosis and cognitive decline, along with cardiometabolic alterations [19,20,21].

### 3.2. Changes in Hormone Levels with Aging in Women

The most dominant hormonal influence on skin aging is estrogen, more specifically 17β-estradiol, which is the most potent sex steroid hormone in humans [21,22,23,24]. It is the major estrogen produced by the ovaries during the reproductive years [21,22,23,24,25,26,27]. The most conspicuous organ in the body that reflects estrogen loss and aging is the skin, especially in women [21,22,23]. The numerous physiological and cutaneous benefits of 17β-estradiol have been reported elsewhere [21,22,23,24,25,26,27,28,29]. In addition, the profile/pattern of estrogen during pre-menopause and fluctuations/decline in perimenopause and postmenopause are covered below.

**Figure 1:**
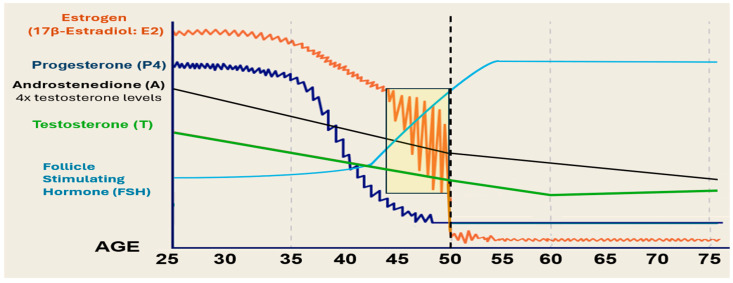
Representative changes in hormone levels with age in women. Representative peak levels of 17β-estradiol in pg/mL during the menstrual cycle are displayed. The yellow highlight box displays wide fluctuations in estradiol levels during perimenopause. [Estrone (E1) in pg/mL is the primary estrogen after menopause and has approximately 1/10 the biological activity of 17β-estradiol]. The other hormones values shown in this figure represent averages of serum levels in ng/mL, except for FSH (in IU/L) from various journal reports [22,23,25,34,35,36] and the Mayo Clinic Laboratory Test Catalog, [https://www.mayocliniclabs.com/test-catalog] accessed on 10 October 2025. Premenopausal progesterone levels during the follicular phase (1 ng/mL), ovulation (12 ng/mL), and luteal phase (2–24 ng/mL) are not shown in the figure above. Adapted and modified from Lephart [37] 2025, vol. 15:1681–1703, with permission from *Dermatology and Therapy* (*Heidelb*) Springer/Nature.

The profile of 17β-estradiol displays maximal levels around the mid- to late 20s in women (Figure 1) [22,23,25]. At around 30 years of age, estrogen levels begin to decline, with corresponding changes in the skin, including the appearance of wrinkles because of the decline in collagen and elastin fibers in the dermal layer [22,23,30,31,32]. Production of 17β-estradiol from ovarian follicles declines further after 35 years of age, with a progressive decline in estrogen levels at 40 to 45 years of age (Figure 1) [21,22,23,26,27,28,29,33]. This interval, called perimenopause, is an adaptive reproductive phase where inconsistent oscillations in ovarian estrogen production occur as follicles respond to gonadotrophin waves of follicle-stimulating hormone (FSH). This occurs until follicles become exhausted and unresponsive, with the onset of menopause, a milestone that in the United States occurs at around 51 years of age, plus or minus 4 years [21,22,23,25,29,33]. After menopause estrogen production occurs not in the ovaries but at peripheral adipose tissue sites, where estrone is produced [21,22,25]. While it is known that skin cells can produce estrogens locally, the activity of the aromatase enzyme, which converts androgens into estrogens, is dramatically reduced to approximately more than 30-times lower than in premenopausal ovarian follicular tissue [22,24]. While estrogens play a leading role in skin, other hormones also have an influence on health and well-being with aging in women [25,34,35,36,37,38].

The profile of progesterone (P4) levels is somewhat different compared to estrogens. Progesterone originates mainly from the ovaries with a small contribution from the adrenal glands, which decreases by about fifty percent by age 40 [25,38] and then continues to decline to very low levels after menopause (Figure 1) [25,38]. The general benefits of progesterone in women include improving (a) sleep quality, (b) mood (alleviates mood swings and anxiety), and (c) cognitive function in some women [21].

Male steroid hormones, androstenedione (A), and the principal androgen testosterone (T), originate from the ovaries and adrenal glands before menopause and then mainly from the adrenals and peripheral tissue sites after menopause (Figure 1) [25]. Androstenedione levels are approximately four times higher than testosterone before menopause, but both androgens are reduced by around fifty percent by age 50 at menopause and continue to decline [25,34]. Although some women may experience very low testosterone levels by 40–50 years of age, the normal range of testosterone in premenopausal women is between 15 and 70 ng/dL or 0.15–0.7 ng/mL. However, one report showed that testosterone levels in women after age 60 years displayed a slight increase suggesting that the loss of estrogens with menopause, along with the negative impact of androgens, contribute to advanced skin aging [35]; notably, this increase in testosterone in aging women needs to be verified. Finally, the general benefits of testosterone in women include improved (a) sexual desire and arousal, (b) energy levels, (c) mood, (d) muscle mass, and (e) bone health [21,38]. For testosterone replacement therapy in menopausal women, the target range is between 50 and 90 ng/dL.

**Figure 2:**
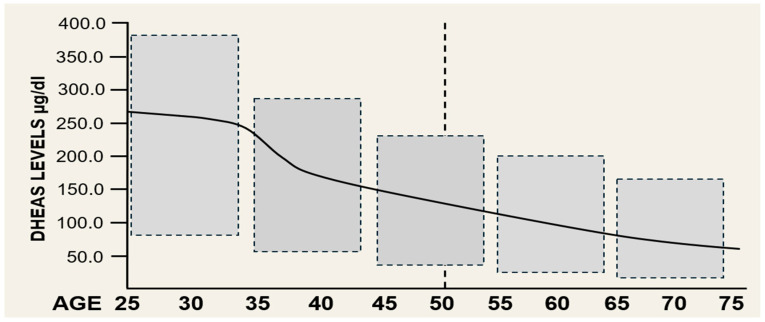
Dehydroepiandrosterone sulfate (DHEAS) levels in adult women with aging. DHEAS is a metabolite of DHEA and is more water-soluble than DHEA. The representative DHEAS values were obtained from the LabCorp website: https://www.labcorp.com/tests/004020/dehydroepiandrosterone-dhea-sulfate accessed on 21 October 2025 The gray-shaded rectangles display the range of DHEAS values by age intervals. For example, the age range 25–34 years had a DHEAS range of 85–380 µg/dL; 35–44 years of age had a DHEAS range of 57–279 µg/dL; 45–54 years of age had a DHEAS range of 41–244 µg/dL; 55–64 years of age had a DHEAS range of 29–221 µg/dL; and 65–74 years of age had a DHEAS range of 20–187 µg/dL.

Finally, dehydroepiandrosterone (DHEA) is a “precursor hormone” that originates from the adrenal glands and ovaries [39,40]. Approximately 98 percent of DHEA circulating in the blood is the sulfated form (DHEAS) [39,40]. DHEAS levels peak in young adulthood and then decline with age [39,40] (see Figure 2). After menopause, DHEAS levels remain low and continue to decline [39,40]. Normal DHEAS levels are a biomarker of healthy adrenal function [39]. The general benefits of DHEAS in women include improved bone and heart health and improved libido, since it is a precursor to testosterone [38,39]. However, as Samaras et al. reported, “DHEA administration is closer to hormonal optimization than hormonal supplementation” [40], and other investigators reporting in systematic reviews do not recommend DHEA in postmenopausal women to improve sexual function and mood or alleviate vasomotor systems [41,42]. Only local DHEA treatment has shown benefits in vulvovaginal atrophy [42]. Thus, “supplementing a pre-hormone is extremely interesting, but is very different from supplementing a hormonal end product”, like 17β-estradiol [40].

### 3.3. The Important Role of Estrogen on Health and Homeostasis via Estrogen Receptors

**Figure 3:**
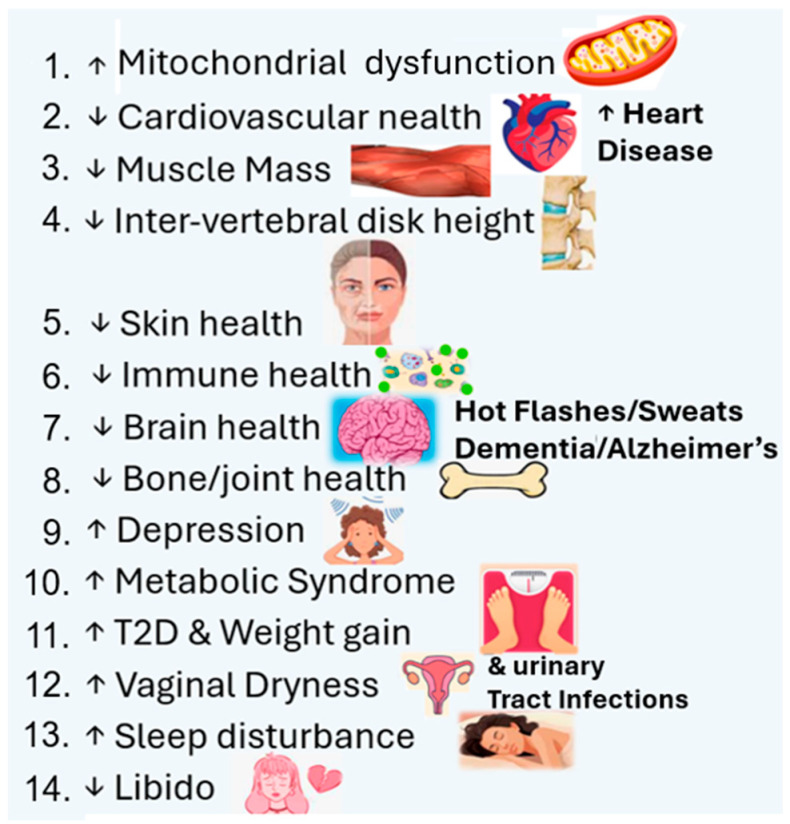
Estrogen deficiency is a pleiotropic factor in the hallmark of aging in women. This figure displays the multi-factor influences of the lack of estrogen as women age, which have been adapted and modified from Lephart [37] 2025, vol. 15:1681–1703, with permission from *Dermatology and Therapy* (*Heidelb*) Springer/Nature. ↑ = increase and ↓ = decrease. One other symptom (not shown) associated with perimenopause is abnormal menstrual bleeding (heavy or irregular periods) [41].

Estrogens have been studied for more than a century. There are three natural estrogens [estrone, 17β-estradiol, and estriol (mainly present during pregnancy)] [22,23,24,25,43]. These compounds are named according to the number of hydroxyl side groups in the molecule [24,43]. All natural estrogens arise by the enzymatic removal of a carbon atom from androgen precursor molecules, a process called aromatization [24,42]. The enzyme is termed aromatase, a product of the CYP19A1 gene [22,26,27,43]. Since virtually every cell in the body expresses estrogen receptors, estrogen actions influence almost all tissues and are responsible for (a) homeostatic regulation, (b) cell proliferation and apoptosis, (c) liver protein expression, (d) lipid metabolism, (e) energy balance, (f) glucose metabolism, (g) immune and cardiovascular alterations, (h) gonadotrophin feedback and gametogenesis, (i) brain neuronal development/memory processing and repair/neurodegeneration, and (j) bone growth [23,43], including estrogen’s positive (agonist) and negative (antagonist) actions on skin, especially in women [22,26,43]. To illustrate the importance of estrogen as a hallmark of aging, Figure 3 displays the pleiotropic impact of estrogen deficiency on 14 different dysfunctions in cellular, metabolic, physiological, and molecular mechanisms [19,20,22,23,36,43,44,45,46].

**Figure 4:**
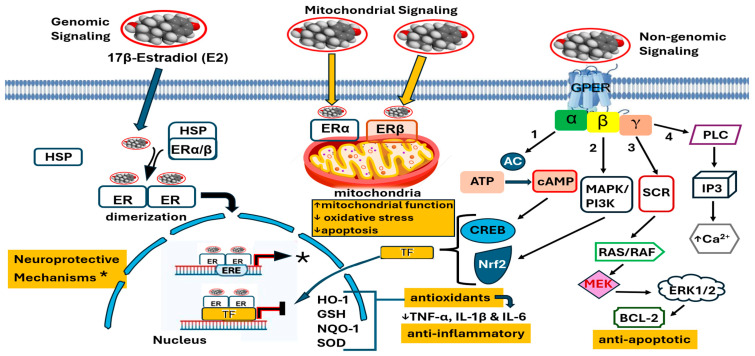
17β-estradiol hormone action via the classical steroid nuclear estrogen receptors ERα and ERβ. ERα and ERβ send their hormonal signals in a slow manner (minutes to hours for activation to occur), while the membrane-bound estrogen receptors (G-protein-coupled seven transmembrane receptor), also known as GPERs, have rapid activation within seconds to minutes due to their binding mechanisms and subsequent activities. ERα and ERβ are also found in the mitochondria, where ERβ predominates. Neuroprotective mechanisms are highlighted in gold rectangles above. The extent to which the genomic, mitochondrial, and non-genomic signaling pathways crosstalk or interact is just beginning to be described. The symbol * represents the genomic signaling pathway of estrogen hormone actions, which includes stimulation of growth and survival factors for proliferation/neurogenesis/neuronal plasticity; anti-apoptotic expression; learning, memory, and cognitive functions; and anti-inflammation. E2 = 17β-estradiol; HSP = heat shock protein; ER = estrogen receptor; ERE = estrogen-responsive element; TF = transcription factors; HO-1 = heme oxygenase-1; GSH = glutathione; NQO-1 = NAD(P)H quinone dehydrogenase 1; and SOD = superoxide dismutase are antioxidants; TNF-α = tumor necrosis factor alpha, IL1-β = interleukin 1-beta, and IL-6 = interleukin 6 are inflammatory molecules; GPER = G-protein-coupled estrogen receptor; G proteins (α, β, γ) subunits; AC = adenylyl cyclase; ATP = adenosine triphosphate; cAMP = cyclic adenosine monophosphate; CREB = cAMP response element-binding protein; MAPK = mitogen-activated protein kinase; PI3K = phosphatidylinositol 3-kinase; Nrf2 = nuclear factor erythroid 2-related factor 2; SCR = Scr family kinases; RAS/RAF = cell signal transduction pathway; RAS = GTPases; RAF = serine/threonine kinase; MEK = mitogen-activated protein kinase; ERK1/2 = extracellular signal-regulated kinase 1/2; BCL-2 = apoptosis regulator; PLC = phospholipase C; IP3 = inositol triphosphate; Ca^2+^ = calcium release from the endoplasmic reticulum. ↑ = increase and ↓ = decrease.

Estrogen receptors are the primary effectors for estrogen action. There are two classical estrogen receptors, estrogen receptor (ER) alpha (α) and beta (β) [22,24,26,36,46] (see Figure 4). Both are nuclear hormone receptors that can act as transcription factors and are similar in structure but share little homology in some of their structural domains (such as the N-terminal and ligand-binding region). Somewhat surprisingly, 17β-estradiol has almost equally high affinity for both ERα and ERβ [24,26,46]. However, when comparing the potency of 17β-estradiol to estrone, 17β-estradiol has a 10-times greater effect compared to estrone and is more than 100-times stronger than estriol [24,47].

Molecular studies have shown tissue-specific and regional expression of ERs in humans. For example, in the human brain, ERα is more highly expressed in the hypothalamus and amygdala, while ERβ is more prevalent in the hippocampus and cortex [48,49]. Both receptors are widely distributed in neurons and glial cells throughout the brain but with distinct regional patterns and functional roles, with ERα linked more to reproductive functions and ERβ to non-reproductive ones [48,49]. Since estrogens penetrate the cell membrane and then bind to classical ERs, with subsequent binding of this complex to DNA within the nucleus, they are referred to as having genomic effects, which are somewhat slow, taking minutes to hours after estrogen exposure [49,50].

### 3.4. Roles of Estrogen Receptors in Neurodegeneration

Both ERα and ERβ play significant roles in neurodegeneration. ERα is crucial for cognitive function, and its decline or genetic variation can increase the risk of cognitive decline and dementia [47,48,49,50,51,52,53]. During menopause, the loss of estrogen and subsequent disruption of ERβ signaling negatively impact brain regions like the hippocampus, leading to cognitive impairment, memory problems, and reduced brain plasticity [48,51,52]. Conversely, maintaining functional ERα is linked to successful cognitive aging and can help protect against age-related cognitive deficits [49,52,53]. ERβ plays a significant role in protecting against cognitive decline, and its decline or dysfunction is associated with an increased risk of cognitive impairment and conditions like AD, particularly in women [49,51,52,53]. ERβ in astrocytes provides protection against hippocampal-dependent cognitive decline, and selective activation in this cell type can mitigate age-related cognitive deficits [48,52,53]. Moreover, evidence from animal studies and observational reports suggest that ERβ and ERβ agonists (see SERM information below) may improve AD symptoms and slow pathology by (a) supporting learning and memory, (b) reducing levels of β-amyloid and inflammation in microglia, which provide immune protection, and (c) activating mitochondrial function disrupted in AD [49,52,53].

Regarding aging in women and brain estrogen receptors, the Mosconi research group utilized positron emission tomography imaging (PET) scans to determine the regional distribution patterns of estrogen receptor expression [54]. The lowest ER density was observed in premenopausal women, followed by perimenopausal, with the high density of ER expression seen in postmenopausal women, which was associated with poorer cognitive/memory performance [54]. This study showed that the loss of estrogen in postmenopausal women displayed the highest ER expression, as if neuronal cells were searching for this chemical steroid signal, which may provide additional support for estrogen replacement therapy to enhance brain health and potentially protect against AD [54].

In addition to classical estrogen receptors, there is a group of estrogen receptors that are tethered to the outer surface of cell membranes [51,55,56,57], which are referred to as membrane-bound estrogen receptors (Figure 4). These estrogen receptors, referred to as GPERs, are composed of a G protein-coupled (with a seven-trans-membrane) structure and, when bound by estrogens, quickly activates (within seconds to minutes) via metabolic pathways (through intracellular second messengers) [50]. Because they do not bind directly to DNA, they have non-genomic effects [50]. While much more is known about ERs (and specifically ERβ), compared to GPERs, some reports have shown positive influences on neurodegeneration via membrane-bound estrogen receptors (GPERs) [55,56,57]. For example, one GPER (G-protein-coupled estrogen receptor 1) plays a significant role in protecting against cognitive decline and is being investigated as a therapeutic target [55]. Furthermore, GPER activation can mitigate cognitive dysfunction by reducing oxidative stress, neuroinflammation, and cell death in neurodegenerative diseases, while promoting neurogenesis [55,56,57]. However, the relationship is complex, with some studies suggesting that higher GPER expression could be linked to faster cognitive decline in specific contexts, like AD, which highlights the need for further research [55,57].

Some compounds that bind to ERs in a targeted and tissue-specific manner, regarding function (agonist or antagonists), are termed selective estrogen receptor modulators (SERMSs) [22,24,25,58]. For example, tamoxifen was the first SERM reported for clinical practice in 1987 in New York, NY, USA, at the First International Chemoprevention meeting [21]. Tamoxifen has unique estrogen-like properties and can act as an estrogen antagonist or agonist depending on tissue type [59]; however, tamoxifen is used to treat breast cancer [59,60]. Consequently, if certain estrogens or estrogenic compounds have a specific affinity for ERβ over ERα, several lines of evidence suggest that ERβ-SERM actions might be beneficial for brain health. For example, SERMs and phytoestrogens (plant-derived estrogenic compounds like isoflavones) show promise for protecting against cognitive decline, but evidence is mixed and more research is needed [61,62,63,64]. SERMs are being investigated for their potential to protect the brain without affecting reproductive tissue, while studies on phytoestrogens have yielded inconsistent results, with some showing potential benefits and others showing no effect or even negative associations, possibly depending on factors like the type of phytoestrogen, dosage, and study population [61,62,63,64].

Additionally, studies that have provided positive results for brain/menopausal parameters include Yao et al. in 2013, who showed that equol, an isoflavonoid compound, displayed potentiation of brain mitochondrial function by increasing genes involved in glucose metabolism and beta-amyloid production and clearance, which may treat menopausal symptoms [65]. In 2015, Utian et al. reported a clinical trial that showed equol (an ERβ agonist), administered twice per day at 10 mg, significantly reduced hot flashes in menopausal women [66]. Similarly, Khapre in 2022, showed that soy isoflavone supplementation decreased menopausal symptoms in peri- and postmenopausal women [67]. Finally, the Brinton/Masconi research group is conducting clinical trials to test an ERβ-targeted nutraceutical, PhytoSERM (NCT06186531, NCT05664477), to determine whether this treatment can activate positive estrogen responses in the brain while inhibiting estrogen actions in the breast, thereby promoting brain health and potentially reducing the risk of menopausal-related depression and AD [68]. Thus, neuroprotective and anti-inflammatory effects of ERβ-agonists or SERMs are being explored, particularly because estrogen-related breast and uterine cancers are mediated through ERα [69].

Finally, ERα and ERβ have been reported to regulate mitochondrial dynamics in the cardiovascular and nervous systems [25,70,71,72,73]. In mitochondria, ERβ, via estrogen hormonal action, stimulates manganese superoxide dismutase to reduce damage from reactive oxygen species (ROS), thereby inhibiting apoptosis in mitochondria [24,58,60,73]. Thus, ERβ activation may support mitochondrial function that involve several pathways, since mitochondrial dysfunction is a key feature in AD.

For example, nuclear respiratory factor 1 (NRF-1), peroxisome proliferator-activated receptor gamma coactivator 1-alpha (PGC-1), endothelial nitric oxide synthase (eNOS), cyclic GMP (cGMP), and mammalian target of rapamycin (mTOR) promote mitochondrial biogenesis through several key downstream targets. Akt (a group of enzymes involved in important cell signaling pathways), often referred to as protein kinase B (PKB), can directly phosphorylate NRF-1, allowing its translocation into the nucleus and binding to promoters of genes that encode mitochondrial proteins, including components of the respiratory chain and mitochondrial transcription factor A (TFAM) [70,72,73]. In addition, Akt controls the nuclear localization and expression of PGC-1, a “master regulator” of mitochondrial biogenesis [70,72,73]. Furthermore, in some contexts, Akt leads to the phosphorylation and activation of eNOS, which increases the levels of cGMP, a signaling molecule that additionally promotes mitochondrial biogenesis [70,72,73]. Finally, Akt can activate mTOR signaling, which stimulates mitochondrial biogenesis by promoting the translation of specific nucleus-encoded mitochondrial proteins [70,72,73]. Thus, estrogen signaling is an important component in defending against AD by supporting brain health in several ways, including protecting neurons, promoting plasticity, and reducing harmful proteins [70,71,72,73].

## 4. Nutritional Factors in Supporting Overall Well-Being and Brain Health

As research increasingly highlights the multifactorial nature of AD, attention has turned toward modifiable lifestyle factors that may help mitigate risk and slow cognitive decline, particularly in postmenopausal women [74,75,76,77]. The hormonal changes accompanying perimenopause and menopause, including the loss of estrogen’s neuroprotective effects, are associated with increased inflammation, metabolic dysfunction, and vulnerability to neurodegeneration [74,75,76,77]. In this context, diet emerges as a critical intervention as specific dietary patterns have been shown to influence brain health through their effects on oxidative stress, insulin sensitivity, and vascular function [74,75,76,77]. Understanding how targeted nutritional strategies can support cognitive resilience provides an important bridge between biological risk factors and practical, non-pharmacological approaches to improving AD outcomes in postmenopausal women [74,75,76], which represents the third pillar presented in this section.

### 4.1. The Mediterranean Diet Versus the Western Diet

**Figure 5:**
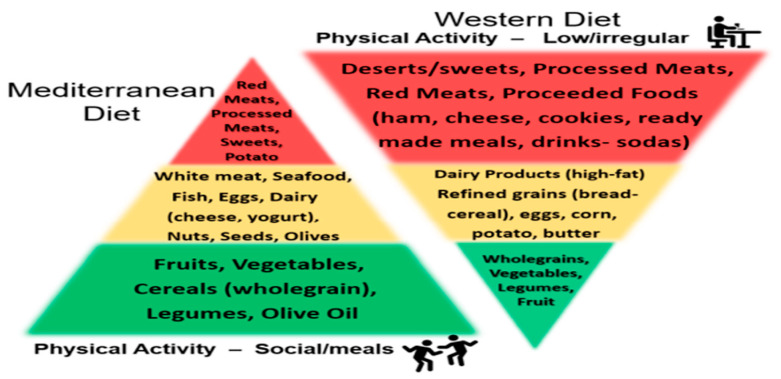
Comparison between Mediterranean and Western diets. Mediterranean Diet: High intake of olive oil (principal source of fat), vegetables (leafy green vegetables, onions, garlic, tomatoes, and peppers), fresh fruits, whole grain cereals, nuts, and legumes. Moderate intake of fish and other seafood, poultry, eggs, and dairy products (mainly cheese and yogurt), low intake of red wine and very low intake of red meat, potatoes, processed meat, refined carbohydrates, and sweets. Western Diet: High intake of pre-packed highly processed foods, red meat, processed meat, high-sugar drinks, candy, sweets, fried foods, conventionally raised animal products (chickens, pigs, turkeys), butter, high-fat dairy products, refined grains, eggs, potatoes, corn (high-fructose corn syrup), and low intake of fruits, vegetables, whole grains, fish, nuts, and seeds. Adapted from Stewart and Lephart (2023) [78], license CC BY 4.0.

While diet has become a focus to enhance human health, attention to which types of diets yield the best outcomes is paramount from the perspective of consumers. The idea of a “Mediterranean diet” (MEDiet) versus the so-called “Western diet” has gained popularity and promise to improve general health status and well-being to address many other diseases and disorders [74,75,76,77,78]. For example, the MEDiet is one of the most widely described and evaluated dietary patterns in scientific literature [74,75,76,77,78]. The MEDiet is characterized by high intakes of vegetables, legumes, fruits, nuts, whole grains, fish, some olive oil, and a moderate intake of red wine, where most proteins and fats are derived from vegetable sources [74,75,76,77,78]. Conversely, the Western diet has a low intake of fruits and vegetables and contains refined carbohydrates, red meat, processed meats, fats, lipids, and cholesterol, all of which increase sympathetic nervous system activity, oxidative stress, and inflammation [74,75,76,77,78] (Figure 5). Additionally Figure 5 shows the relative levels of physical activity associated with the two diets.

### 4.2. The Mediterranean Diet and Alzheimer’s Disease

The first clinical study on the MEDiet and the risk of AD was published in 2006 by Scarmeas et al., where the authors concluded that a higher adherence to the MEDiet was associated with a reduction in the risk of AD [79]. Prior research in AD had focused on individual dietary components [79]. Since 2006, more than four hundred clinical studies have been published on the association between the MEDiet and AD. While a large body of evidence comes from observational studies that demonstrate a strong association between adherence to a MEDiet and a reduced risk of AD and slower cognitive decline, there are also several interventional studies and randomized controlled trials (RCTs). In addition, various systematic reviews and meta-analysis reports have been published on the role of the MEDiet in reducing the risk of cognitive impairment, dementia, and AD [80,81]. In general, these reviews highlight the essential role of dietary interventions in the prevention and management of dementia and AD and therefore offer insights into which diet may impact brain health [80,81,82,83].

#### How the Mediterranean Diet Helps Brain Health

The MEDiet may help brain health via several possible, non-exclusive mechanisms.

(a) Many studies have shown that adherence to the MEDiet is linked to fewer signs of AD pathology, including reduced amyloid and tau protein buildup [83,84,85,86,87,88,89]. For example, Ballarini et al. in 2021 found that the MEDiet was protective against memory decline and mediotemporal atrophy, with an associated decrease in amyloidosis and tau pathology [87], confirming and extending the findings of previous reports [84,85,86,87,88,89].
(b) The MEDiet may potentially offset genetic AD risk—where the diet’s protective effects are pronounced in individuals with a high genetic risk for AD, such as those carrying two copies of the apolipoprotein E4 (APOE4) allele, a variant that is associated with increased risk of developing dementia [90]. This study by Liu et al., indicated that “these findings suggest that dietary strategies, specifically the MEDiet, could help reduce the risk of cognitive decline and stave off dementia by broadly influencing key metabolic pathways” [90]. However, a limitation of this study was that the cohort investigated consisted of well-educated individuals of European ancestry, and more research is needed in diverse populations. In addition, the study by Liu (2025) [90] extended a previous study in 2023 by Shannon et al., which showed that adherence to the MEDiet was associated with lower dementia risk, but this study did not demonstrate clear evidence of interaction with genetic risk [91].
(c) Brain imaging biomarkers such as (larger brain areas/volume and increased cortical thickness measured via MRI, and biochemical markers via PET scans) have shown that critical brain areas for memory and function are associated with better cognitive performance in subjects on a MEDiet [80,81,83,86,92,93,94,95]. Some studies that utilized MRI-based biomarkers for AD showed that adherence to the MEDiet not only increases cortical thickness but also improves insulin sensitivity [93,94]. In a meta-analysis of MRI results examining the MEDiet and dementia showed reduced white matter hyperintensities (or bright spots on brain MRI that indicate damage) in older adults on the MEDiet [94]. In a study that utilized PET scans in middle-aged adults (30–60 years of age) over a three-year interval, the effects of high or low adherence to a MEDiet on AD biomarkers (brain β-amyloid load and neurodegeneration via glucose metabolism) were monitored. Low adherence resulted in reduced glucose metabolism and higher β-amyloid deposition, whereas high MEDiet adherence was estimated to provide 1.5–3.5 years of protection against AD [88]. Finally, in 2018, Sindi et al. reported that healthy dietary changes in midlife may be associated with reduced dementia risk later in life. The study followed a cohort of 2000 individuals (mean baseline age = 56 years), with follow-ups at two later life examinations at mean ages of 70 and 78 years [95]. The authors highlighted the importance of dietary components (fats, vegetables, sugar, and salt), which may have synergistic effects in reducing dementia risk [95,96].
(d) The potential mechanisms of high MEDiet adherence include the antioxidant and anti-inflammatory effects of dietary compounds by activating cell signaling and molecular pathways. The abundance of antioxidants in the MEDiet and its potential to reduce oxidative stress have been clearly established [96,97]. Furthermore, other benefits of the MEDiet have been reported, as summarized below.

For example, Nuclear Factor Erythroid 2-Related Factor 2 (Nrf2) is a cellular defense mechanism that protects against oxidative stress by activating the production of antioxidant and detoxification enzymes [98]. Under normal conditions, the repressor protein Keap1 binds to Nrf2 in the cytoplasm, marking it for degradation. When the cell is stressed, Keap1 releases Nrf2, which then translocates to the nucleus and binds with the antioxidant response element (ARE) to turn on protective genes [85]. MEDiet compounds in various foods can also inhibit Nuclear Factor kappa-light-chain-enhancer of activated B cells (NFk-B), which is involved in immune response and inflammation [98,99,100]. In this regard, NFk-B is thought to trigger caspase cascades and programmed cell death in mitochondria [99], which may be involved in neuronal dysfunction or damage via beta plaques and tau tangles [101]. Other benefits of the MEDiet included improved cardiovascular health, better blood sugar and lipid metabolism, and enhanced gut microbiome [102,103]. While direct studies on the effects of the MEDiet on human glymphatic clearance are limited, research highlights several indirect mechanisms through which it supports waste removal [104,105]. However, investigators have shown that β-carotene concentrations in serum were associated with better neurological volumes and parameters, most likely due to antioxidant activity maintaining the glymphatic system function of the brain [106]. Specifically, the glymphatic system is a unique clearance pathway in the brain that relies on cerebrospinal fluid (CSF) and glial cells to remove metabolic waste and other harmful substances and is an emerging therapeutic approach for neurological disorders [107].

## 5. Neuroinflammation with Aging: Estrogen Decline/Loss and Nutritional Influences

Neuroinflammation emerges as a central hallmark of brain aging, and its progression in women is increasingly recognized as being shaped by the convergence of hormonal and nutritional factors [1,2,8,19,20,21,104,105]. The decline in estrogen that accompanies menopause removes a critical modulatory influence on microglial activation, cytokine production, and oxidative stress, thereby amplifying age-related inflammatory signaling within the central nervous system [41,49,52,92,93,98,99,100]. At the same time, nutritional status across midlife and later years—particularly intake of anti-inflammatory nutrients, antioxidants, and metabolic substrates—can either exacerbate or buffer these neuroimmune changes [102,103,104]. In postmenopausal women, the intersection of estrogen loss with dietary patterns and nutrient availability creates a distinct biological context in which neuroinflammatory processes may be accelerated or mitigated underscoring the importance of integrated hormonal–nutritional frameworks for understanding brain aging trajectories [106,107], which is the fourth pillar presented in this section.

### 5.1. Peripheral Inflammation Is Associated with Neuroinflammation

**Figure 6:**
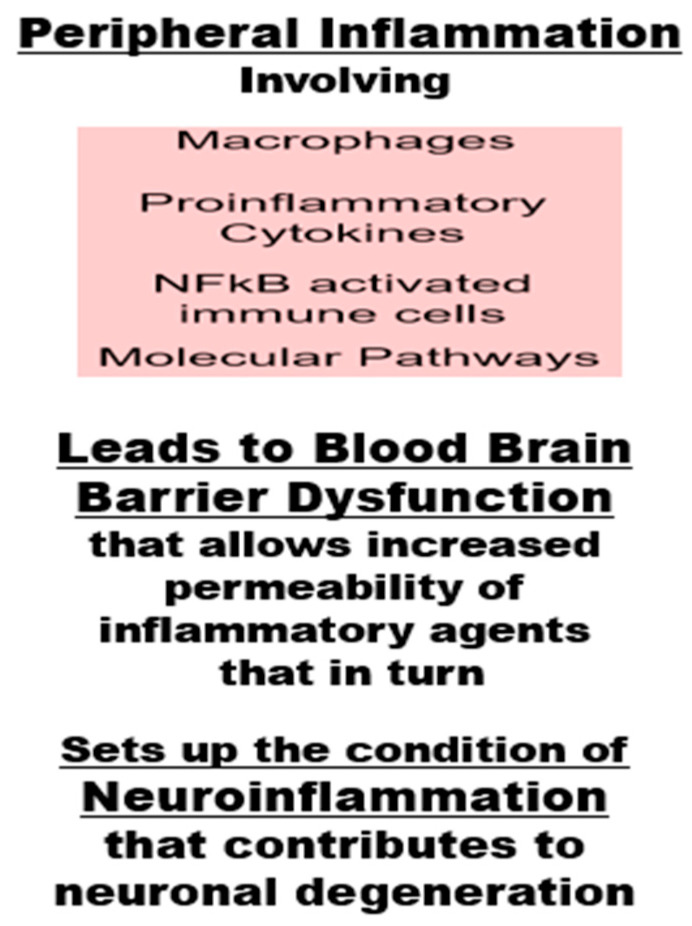
Mechanism between peripheral inflammation and neuronal degeneration. Peripheral inflammation resulting from pro-inflammatory cytokines and immune cell activation (**top panel**) causes dysfunction of the blood–brain barrier (BBB), allowing increased permeability of inflammatory agents into the brain and neurons (**middle panel**), which in turn sets up the condition of neuroinflammation and contributes to neuronal degeneration (**bottom panel**). This process defines the cascade mechanism of neuroinflammation and the hallmarks of many brain diseases and disorders.

Neuroinflammation occurs when the blood–brain barrier (BBB) becomes compromised by peripheral inflammation, allowing pro-inflammatory molecules and immune cells to enter the brain and trigger a response from the brain’s own immune cells, such as microglia [108,109]. This neuroinflammatory environment can then lead to neuronal damage and loss, a process observed in neurodegenerative diseases like AD, Parkinson’s disease, and amyotrophic lateral sclerosis [108,109]. Further, developing evidence suggests that an imbalance in gut microbiota in the gut–brain axis (a bidirectional communication pathway linking the gut and the brain) can lead to neuroinflammation and play a key role in BBB integrity [110,111]. In brief, gastrointestinal dysbiosis and chronic inflammation participate in BBB disruption through immune chemical messengers and various chemical and molecular pathways [110,111,112]. For example, peripheral inflammation with increased pro-inflammatory cytokines and immune cell activation results in dysfunction of the BBB [110,111]. The altered BBB structure and function allow pro-inflammatory molecules to enter the brain and cause neuroinflammation, which contributes to neuronal degeneration by the accumulation of altered proteins, amyloid plaques, synaptic loss, and increased oxidative stress [108,109,110,111]. These are hallmarks of neurodegenerative diseases like AD, Parkinson’s disease, and amyotrophic lateral sclerosis (see Figure 6) [108,109,110,111,112].

### 5.2. Neuroinflammation and Neurodegeneration

The immune system and the nervous system are intricately connected via chemical and molecular pathways through which they communicate with one another [113]. In acute inflammation, there is local or regional activation of immune cells at the site of infection or trauma, with the removal of pathogens over a short period of time, enabling cell and tissue repair and a return to homeostasis [113,114]. Conversely, chronic inflammation is characterized by immune cell activation and recruitment of downstream pro-inflammatory compounds, resulting in off-target effects that can lead to altered cellular viability, function, and tissue damage [113,114].

Neuroinflammation is defined as a complex biological response of the central nervous system (CNS, i.e., brain and spinal cord) to damage from toxins, trauma, infection, aging, oxidative stress, chronic stress, or autoimmunity [113,115,116]. This is characterized by the activation of microglia and astrocytes leading to the production of pro-inflammatory chemical messengers such as cytokines and chemokines and to alterations in CNS homeostasis, which can result in neuronal degeneration [113,115,116].

The neuroimmune system is primarily composed of microglia, oligodendrocytes, and astrocytes [113,114,115,116,117,118,119,120]. Microglial responds to signs of injury, infection, oxidative stress, or altered cellular function and act as CNS resident macrophages (differentiated from hematopoietic myeloid progenitor cells) [113,117]. Microglial help regulate brain development, neuronal network maintenance, and injury repair and are important mediators of neuroinflammation [121]. Microglial have two phenotypes, M1 and M2, where M1 microglia are pro-inflammatory and release inflammatory cytokines like interleukin (IL-6) and tumor necrosis factor alpha (TNF-α), along with many other pro-inflammatory compounds [113,117]. In contrast, M2 microglia are anti-inflammatory and produces anti-inflammatory cytokines such as IL10 (among other anti-inflammatory molecules), which clean up the CNS via phagocytosis of debris and promote tissue repair [113,117]. Once activated, microglia become mobile and remain at the site of infection or injury for a long time (weeks to months or longer with chronic stress/infection or oxidative stress), releasing cytokines and neurotoxic agents that can cause CNS damage [113,115,122].

Neuroinflammation increases with age due to a “pro-inflammatory state” characterized by heightened microglial activation, increased pro-inflammatory cytokines, and decreased anti-inflammatory molecules. This chronic, low-grade inflammation, sometimes called “inflammaging,” primes the brain to be more susceptible to stressors and is linked to age-related conditions like memory impairment and neurodegenerative diseases [123,124].

Oligodendrocytes play a complex role in neuroinflammation by sensing and responding to inflammatory signals, but their actions are not always beneficial [118,125,126,127,128]. They can actively contribute to inflammation by producing pro-inflammatory cytokines and chemokines and by expressing molecules like MHC-I and MHC-II that present antigens and activate other immune cells, like T cells [118,125,126]. Conversely, they can also release anti-inflammatory factors, and their inflammatory responses are often reactions to pro- or anti-inflammatory signals initiated by microglia [113,117,125]. Hence, some reports suggest that oligodendrocytes drive neuroinflammation [128,129], while other reports suggest neuroprotective effects [118,125,127,129,130,131].

The last major glial neuroimmune cells are astrocytes. In general, astrocytes help form the physical structure of the brain and extracellular matrix [113,117,118]. Astrocytic “endfeet” processes closely interact with blood vessels, helping to form and maintain the BBB, which regulates the passage of substances from the blood into the brain [113,117,118]. Astrocytes are crucial for brain energy metabolism because they store glucose as glycogen and utilize lactate as an energy source for neurons [107,111,112]. They also provide neurotransmitter homeostasis by recycling neurotransmitters for synaptic transmission [113,117,118] and regulate ion and fluid homeostasis [107,111,112], which help control blood flow, maintain CNS homeostasis via clearance of cellular debris, regulate neurotransmitter levels, and maintain the BBB [113,127,128]. Reactive astrocytes mount diverse responses that contribute to tissue repair and neuroprotection via the STAT3 pathway or promote neuroinflammation through the release of various pro-inflammatory compounds via the NFkB pathway [113,117,132].

**Figure 7:**
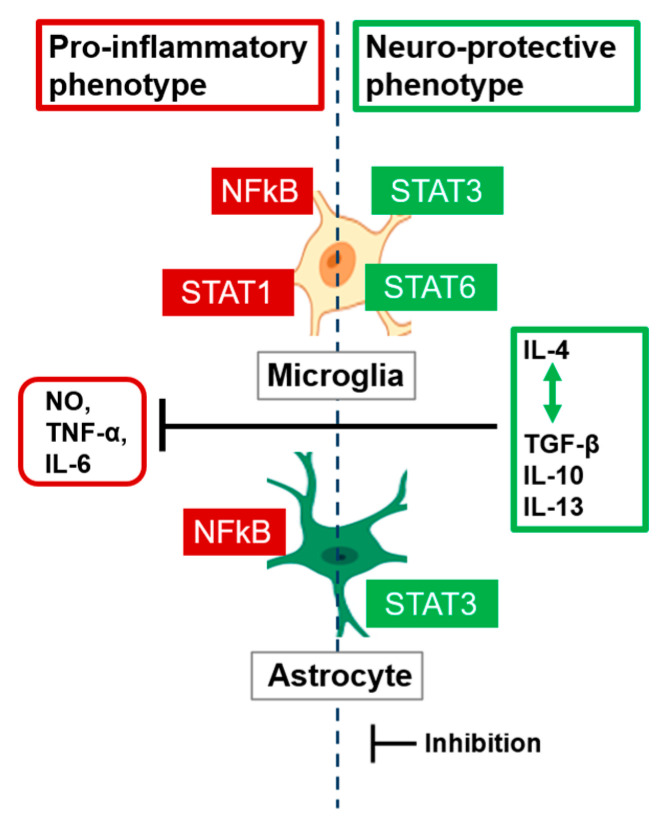
Biochemical and molecular signals involving microglia and astrocytes. The pro-inflammatory microglia are activated by IFNs and LPS via the activation of NFkB and STAT1, which then release many pro-inflammatory factors displayed in red. The neuroprotective microglia by the activation of STAT3 and STAT6 release several neurotrophic factors and anti-inflammatory agents shown in green. The activation of NFkB induces pro-inflammatory astrocytes shown in red. The activation of STAT3 in astrocytes induces neuroprotective astrocytes that release several neuroprotective compounds shown in green, which can suppress or inhibit NO, TNF-α, and IL-6.

This brief overview demonstrates the importance of microglia and astrocytes in neuroinflammation and neurodegenerative disorders, such as AD, Parkinson’s disease, and amyotrophic lateral sclerosis, as key regulators of inflammatory responses in the CNS, which are displayed in Figure 7. In fact, there are more than six hundred brain diseases, and neuroinflammation plays a role in nearly all of them [132].

### 5.3. Neuroinflammation and Estrogen Decline and Loss

Changes in and loss of estrogen levels as women age have a dramatic impact on neuroinflammation due to estrogen’s neuroprotective effects, which include anti-inflammatory and antioxidant factors, maintenance of BBB integrity, and immune cell regulation [52,53,133,134,135]. The decline in estrogen actions can lead to increased neuroinflammation and susceptibility to neurodegenerative disorders like AD and Parkinson’s disease, as well as age-related cognitive decline and mood changes [52,53,133,134,135,136,137].

**Figure 8:**
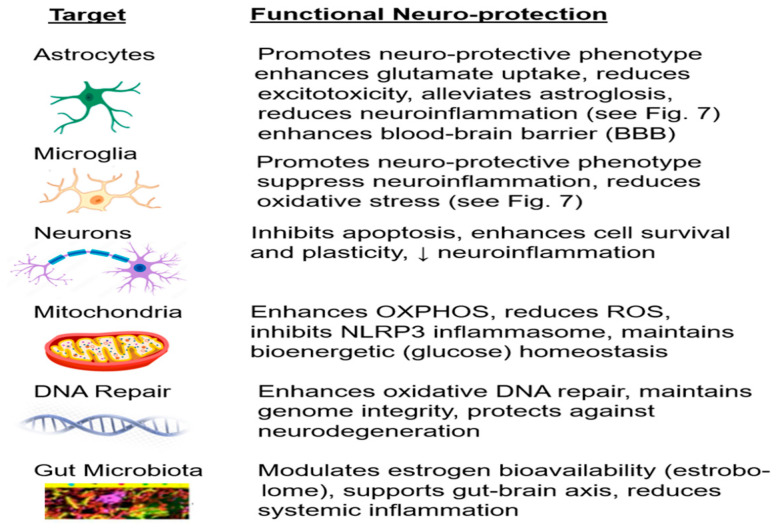
Estrogens (17β-estradiol) neuroprotective effects in target cells, organelle, DNA, and organ system. ↓ = decrease.

The mechanisms of estrogen’s anti-inflammatory actions are more prominent in younger women, when estrogen levels are higher but decline with aging or in those with long-term hormone deprivation [133,134,135,136,138]. For example, estrogen can reduce the production of inflammatory molecules such as cytokines (e.g., IL-1β, IL-6, and TNF-α). Concurrently, estrogen receptor activation (mainly via Erα, GPER1, and sometimes ERβ) can suppress pro-inflammatory signals and pathways such as the nuclear factor-kB (NFkB) pathways [136,138], which is a central regulator of inflammation and orchestrates the expression of pro-inflammatory genes as well as the innate and adaptive immune responses [139,140]. In addition, the nucleotide-binding oligomerization domain-like receptor protein 3 (NLRP3), a triggering component of inflammasome formation that initiates the release of pro-inflammatory cytokines, is activated under conditions of estrogen deficiency [137,141]. Additionally, estrogens increase the activity of antioxidant enzymes, which protect neurons from damage [133,134,135,136]. However, without sufficient estrogen, the brain is more vulnerable to oxidative stress, which is a key contributor to neuroinflammation [132,133,134,135,136,138,139]. During brain aging, estrogen has DNA repair properties by stimulating growth factors such as brain-derived neurotrophic factor (BDNF), a critical factor in synaptic plasticity that declines after menopause [134,135,136,137,138,139,140]. Moreover, estrogen helps maintain the structural soundness of the BBB, while the decline in estrogen can make the BBB more permeable, allowing inflammatory molecules and other harmful substances to infiltrate the brain [132,133,134,135,137,138]. Estrogen can modulate the behavior of immune cells like microphages and microglia, often promoting an anti-inflammatory state. However, the decline and loss of estrogen in aging women is linked to increased risk of neurodegenerative diseases, with neuroinflammation being a significant factor for AD and Parkinson’s disease, along with other brain disorders [6,133,134,135,136,141,142,143,144]. This brief section underscores estrogen’s key role in modulating neuroinflammation through multiple mechanisms (see Figure 8).

### 5.4. Neuroinflammation and Nutrition (Lifestyle Factors)

Several factors contribute to increased neuroinflammation, many of which are related to lifestyle and environmental conditions, such as air pollution [145,146], poor quality of sleep [147,148], chronic stress (smoking and vaping) [147,148,149], sedentary behavior and/or lack of exercise [147,148], and poor and/or inadequate nutrition [146,147,148,149]. Neuroinflammation is increasingly implicated in a range of brain diseases and disorders, including neurodegenerative diseases (like AD), mood disorders (anxiety and depression), and poor cognitive function [147,148,149,150]. However, this section focuses on nutrition as a factor in neuroinflammation.

For aging women, nutrition is a critical, modifiable factor that directly influences the risk of neuroinflammation, age-related cognitive decline, and potential neurodegenerative disorders [147,148,149,150]. Pro-inflammatory diets common in Western cultures exacerbate this risk, while anti-inflammatory diets, such as the MEDiet, offer potential neuroprotective benefits [146,147,148,149]. There is a heightened susceptibility to neuroinflammation when estrogens decline with perimenopause and with the loss of 17β-estradiol at menopause [149]. For example, Bernier et al. in 2025, reported that middle-aged women may face a vulnerable health state where Western dietary patterns already linked to inflammation reinforce systemic inflammation involving the gut–brain axis [149]. Moreover, peripheral inflammation may cross the BBB, promoting neuroinflammation and oxidative stress and enhancing neurodegenerative changes, especially in women with obesity and diabetes [149]. Other researchers report that diet can act as a modulator of inflammatory process in neurological diseases, where sugar, ultra processed foods, and high-fat intake can have detrimental effects on brain health and neuroinflammation, while oxidative stress can damage lipids, proteins, and DNA [149,150,151]. In contrast, anti-inflammatory foods like fruits and vegetables, omega-3, healthy fats (olive oil, nuts, and seeds), and whole grains have neuroprotective effects and are associated with lower levels of inflammatory biomarkers, such as C-reactive protein (CRP) and IL-6 [149,150]. Additionally, Singhaarachchi et al., in 2024, examined how dietary lipid profiles and polyunsaturated fatty acids may regulate the inflammatory process leading to AD [152]. Finally, the relationship between dietary habits and lifestyle factors have been examined to reduce neuroinflammation and AD, particularly in aging women [147,148,149,150,152,153]. For instance, studies have examined microglia and astrocyte activation, pro-inflammatory cytokines, TNF-α, chemokines, Toll-like receptor signaling pathways, the gut–brain axis, dietary food components, obesity, diabetes, alcohol consumption, smoking, lack of sleep quality, and physical exercise as contributors to neuroinflammation and neurodegeneration [147,148,149,150,152,153].

## 6. Discussion

### 6.1. Cognitive Decline with Aging and the Loss of Estrogen

**Figure 9:**
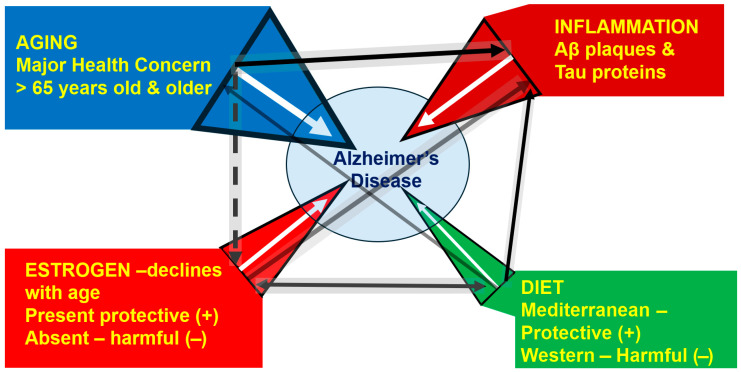
Outline of four pillars—aging, estrogen, nutrition, and inflammation—that may contribute to the onset and progression of AD. The size/area of the colored triangles and white arrows for each of the four pillars (aging, inflammation, estrogen, and diet) represent the percentages of each factor that may contribute to the onset and progression of AD via biological network analysis.

Although age is the primary risk factor for AD [7,148,149], AD is not a normal part of aging (Figure 9). In the United States, the prevalence of AD in individuals aged 65–74 is approximately 5 percent; at ages 75–84, prevalence is approximately 13 percent; at ages 85 and older, the prevalence of AD is approximately 33 percent; and over 95 years of age, prevalence is approximately 50 percent [8]. Brain neurons of individuals with AD feature aging hallmarks, including genomic destabilization, decreased telomere length, alteration in epigenetic signatures, and mitochondrial dysfunction associated with oxidative stress, as well as many other defective structural and functional components [154,155]. There appears to be a convincing relationship between sex and AD risk, since almost two-thirds of Americans with AD are women (where 4.4 million women aged 65 and older have this disorder), which may be due a combination of biological factors like changes in hormone levels, genetics, and lifestyle factors [8,156]. Since women face a disproportionate burden of AD relative to men, this suggests that neuropathological hallmarks of AD are laid down beginning in midlife [154,155,156,157,158].

In this regard, some investigators have reported that AD-related brain changes develop during a 10–20-year prodromal interval that originates during midlife, which for women corresponds to the proximate change in sex hormones, especially the decline in estrogen during perimenopause and the loss of 17β-estradiol from the ovaries at menopause [156,157]. Preclinical evidence for the neuroprotective effects of estrogen strongly supports the associations between the decline and loss of estrogen and the risk of AD [19,23,43,52,53,157,158]. Along with estrogen’s significant health-protective actions outlined in Section 3, estrogen receptors, via a variety of biochemical and molecular pathways, inhibit neurodegeneration by protecting neurons, promoting neuroplasticity, reducing oxidative stress, enhancing mitochondrial function, and reducing the harmful impact of detrimental protein components [22,23,26,27,28,29,30,31,32,43,44,45,46] (Figure 9). Additionally, recent reports demonstrate that estrogens have other actions that may also contribute to the sex disparity observed in AD, such as olfaction, sleep, and glymphatic function [159,160]. In fact, Sahin and Yildirim, in 2025, showed that the loss of estrogen (along with progesterone) amplifies microgliosis, NF-kB activation, cytokine release (IFN-γ, IL-6, IL-8), and aquaporin-4 (AQP4) mis-localization, whereas physiological hormone replacement reverses these changes, restores AQP4 polarity and stabilizes the blood–brain barrier [160]. Notably, estrogen enhances AQP4-mediated water transport at astrocytic endfeet, increasing glymphatic clearance, which is a process recognized as a determinant of brain health and resilience to neurodegeneration [160]. The authors concluded that estrogen loss, in a sex-dependent manner, decreases the regulation of AQP4 and glymphatic flow, serving as plausible contributors to the increased incidence and faster progression of AD in postmenopausal women [160].

However, the consequences of estrogen decline and loss examined in epidemiological and hormone replacement studies have produced mixed or negative results [161]. In the wake of the Women’s Health Initiative (WHI) study results released in 2002, there was a substantial contribution to the subsequent trend of fear and avoidance of hormone replacement therapy (HRT) [more commonly referred to as menopause hormone therapy (MHT) [161]. However, in this presentation, the term HRT is used throughout the text]. Following the aftermath of the WHI publications, millions of women over more than a generation have been deprived of the positive effects of HRT [162,163,164,165]. The WHI faced methodological limitations due to its use of older populations (generally women in their 60s and 70s and did not mainly study early postmenopausal women), the use of a combined estrogen–progestin (conjugated equine estrogen-CEE + medroxyprogesterone acetate -MPA) regiment that confounded results with progestin’s negative effects (unlike estrogen alone), lack of generalizability due to selected, healthier participants, and potential biases in risk assessment (family history for cancer), leading to misinterpretation and overstated fears about HRT [162,163,164,165,166]. Thus, the WHI failed to differentiate risks by age (timing), hormone type, or baseline health [162,163,164,165,166]. In several re-analyses of the WHI study, the corrected absolute risks of adverse events were much lower than previously thought [166,167]. For example, HRT appears to be cardioprotective [167] and protective against dementia [168], especially for the management of vasomotor symptoms, which demonstrate a symptom reduction of 60–90 percent with HRT [169]. In addition, while CEE and MPA were the predominate hormones utilized in the early years of HRT, these interventions have been largely superseded in the last 20 years by regulated, approved formulations of estradiol, along with various later-generation progestogens and progesterone preparations [169]. In fact, when the WHI results were re-analyzed based on the smaller subgroup of women (those fifty to fifty-nine years of age) in a systematic review and meta-analysis reported in *JAMA*, the results showed that HRT started in midlife was apparently associated with a reduced risk of dementia [170], which has been supported by several observational studies [171,172].

Recently, two reviews from the Mosconi laboratory raised awareness of ovarian steroid hormones as a critical factor in brain aging and AD [173,174]. In the first review, published in 2022, Mosconi et al. examined the contribution of estrogens in maintaining cognition in women, which has also been reported by other investigators, and provided convincing evidence implicating the menopause-related decline in 17β-estradiol in cognitive aging and AD risk [173]. Specifically, their report suggested that hormonal changes associated with menopause can trigger a “bioenergetic crisis” in the female brain, creating a window of opportunity for early signs of AD pathology to emerge, as examined by brain imaging (MRI and PET scans) looking at the presence of increased amyloid-beta deposition and altered glucose metabolism during the menopausal transition (starting in midlife) [173].

The second review by Mosconi et al., published in 2023, reported a systematic meta-analysis of the effects of menopause hormone therapy on AD risk and dementia [174]. This review examined six randomized clinical trials with 21,065 treated and 20,997 placebo participants, as well as 45 observational studies (768,886 patient cases and 5.5 million controls), where the lead author stated, “hormones work best for the brain when taken in midlife in the presence of menopausal symptoms to support women through the menopause condition, which may represent a window of opportunity” for HRT [174]. The length of time of HRT was an important factor, with a 26 percent reduction in dementia risk if MHT was taken for more than 10 years [174]. Thus, overall, these findings provide new insight into the association of HRT use and AD incidence and support the renewed interest in evaluating HRT for AD and dementia risk reduction [174].

Finally, Figure 9 outlines the different aspects (aging, estrogens, nutrition, and inflammation) that may contribute to the onset and progression of AD, as covered in this section.

1. The single greatest risk factor is aging, where AD represents approximately 70% of all dementia cases. 2. Neuroinflammation, while not a primary “trigger”, is a core pathological driver of neuronal damage. 3. Estrogen, a modifiable hormone factor that declines with age significantly impacts on women’s higher AD risk of onset and progression; HRT may be protective if started early, though complex in its application. However, since reductions in estrogen are known to increase inflammation, this pillar may play a greater role than displayed in this network analysis. 4. Diet is an important, modifiable lifestyle factor that works indirectly to mitigate AD risk and enhances general health and well-being with aging (by decreasing obesity and type 2 diabetes/metabolic syndrome), and like the declination in estrogen, may have a greater role than displayed above. The size of the black arrows represents the impact of each pillar and/or their potential synergistic effects.

### 6.2. Hypothesis: Should Women During Perimenopause Be Administered Estrogen?

**Figure 10:**
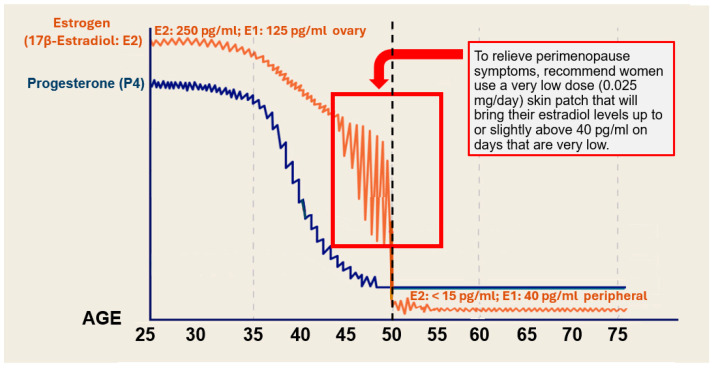
Proposed hypothesis of administration of estradiol during perimenopause. See text above for details. A 40 pg/mL maintenance dose would not influence the very high levels of estradiol but would maintain systemic levels to address perimenopausal symptoms and potentially protect against cognitive decline observed after menopause. Adapted and modified from Lephart [37] 2025, vol. 15:1681–1703, with permission from *Dermatology and Therapy* (*Heidelb*) Springer/Nature.

Menstruation can stop for intervals of varying lengths during perimenopause due to irregular ovulation caused by declining hormone levels, leading to unpredictable cycles that can become longer or shorter [22,23,25]. These changes are a normal part of the menopausal transition; however, it is also important to remember that during perimenopause, menstruation may stop for certain intervals [22,23,25]. Conversely, there are wide fluctuations in estradiol production from the ovary, where levels can be very high one day and very low on another day (Figure 10) [22,23,25]. This is due to the intense stimulation of FSH on the aging ovarian follicles, resulting in a roller coaster pattern of estradiol production. During perimenopause, very low levels of estradiol are associated with symptoms such as (a) hot flashes and night sweats, (b) palpitations (rapid heartbeat), (c) mood swings, (d) sugar cravings, and (e) anxiety [21,22,23,26,27,28,29,33]. Perimenopause lasts, on average, four to eight years [21]. To relieve these perimenopause symptoms and address AD prevention, a hypothesis is presented suggesting that women may use a very low-dose (0.025 mg/day) skin patch that will bring their estradiol levels up to or slightly above 40 pg/mL (Figure 10). This will not only address the lack of adequate estradiol levels but prevent future symptoms as menopause approaches and provide potentially protective treatment for all the positive actions estrogen has in many tissues/organs (see Figure 3), especially for cognitive decline and possibly for ameliorating the onset of AD as women transition through menopause [175]. This is especially important since the US FDA recently announced that it is reversing a 2003 decision that put stringent warnings on hormone therapy products for menopausal women, saying that these treatments offer heart, brain, and bone health benefits [176]. In reference to neurodegenerative diseases, this hormonal treatment may slow down the risk of Alzheimer’s disease in women, since a generation of females have been deprived of hormonal/menopausal therapy that may decrease aging, estrogen loss, and neuroinflammation, thereby enhancing health and well-being. However, it is important to note appropriate safety monitoring protocols, such as breast cancer screening for hormone-sensitive cancers and other contraindications such as active liver disease, active or recent thromboembolic disease, or heart disease, as well as other personal health history parameters, should be in place. Finally, the North American Menopause Society (NAMS) released updated guidance in 2025, emphasizing individualized hormone therapy initiated within 10 years of menopause or before age 60 for the best benefit–risk stressing shared decision-making and highlighting vaginal hormones as first-line for Genitourinary Syndrome of Menopause (GSM). Key points include prioritizing transdermal hormone therapy for lower risks (venous thromboembolism/stroke), continuing hormone therapy beyond age 65 if beneficial (with evaluation), and using vaginal estrogen for GSM even with breast cancer history [177].

In this regard, the Masconi research group published in 2025 that, while randomized clinical trials (RCTs) conducted in midlife are lacking, observational research provides evidence that time-sensitive AD risk reduction is key in making clinical decisions concerning HRT [175]. Thus, potential lower dementia risk may warrant consideration to use HRT, since other existing therapies for AD lack preventive efficacy [175].

Although there has been a paradigm shift on the use of HRT, surveys show women fear using HRT due to concerns about side effects like an increased risk of breast cancer and cardiovascular disease, fueled by the misinterpretation of early studies like the Women’s Health Initiative (WHI) from 2002 [162,163,164,165,166,178]. Other reasons for fear include being advised against it by doctors or receiving negative information from media, family, and friends, leading to widespread avoidance of the therapy even when it could be beneficial for managing menopausal symptoms [162,163,164,165,166,178]. Associated with this fear and confusion about the use of HRT is that some women will consider taking HRT but want the steroids (estrogen, progesterone, etc.) to be derived from plant sources (bio-identical hormones) [179]. Finally, this confusion and fear have driven the use of phytoestrogen and polyphenolic compounds that, in general, are ERβ agonists to treat menopausal symptoms [180,181]. Positive effects of phytoestrogens on postmenopausal health have also been reported [180,181,182,183]. However, since phytoestrogens are considered endocrine disruptors, this also adds to the fear and confusion, as well as misinformation and controversy of using plant-based products for women’s health [184].

### 6.3. Nutrition and Neuroinflammation in Cognitive Decline

Section 4 above covered the importance of nutritional factors in supporting overall well-being and brain health, including peripheral inflammation that can lead to neuroinflammation.

As mentioned previously, nutrition for cognitive function involves a balanced diet rich in fruits, vegetables, whole grains, lean proteins, and healthy fats, while limiting processed foods, sugar, and saturated fats. Key nutrients like omega-3 fatty acids, flavonoids, vitamins (B, D, K, and C), and antioxidants are crucial for supporting brain health and function [185] (Figure 9).

It has been proposed that young adulthood and middle age are crucial time intervals for determining cognitive health in old age [185]. Therefore, the target populations are people in their 40s and 50s, before they develop dementia [174]. Adherence to an anti-inflammatory diet, like the MEDiet, is associated with lower AD mortality and modified risk factors such as cardiometabolic health, obesity, hypertension, smoking, physical inactivity, and sleep quality represents a cognitive health perspective associated with positive outcomes [186].

In this regard, Toups et al., in 2022 [187], reported a pilot study with 25 patients (13 females and 12 males) with AD or mild cognitive impairment, aged 50–70, who were treated for nine months with a personalized medicine protocol. This protocol included a health coach, nutritionist, physical trainer, and physician, where their diet was plant-based and high-fiber; exercise included aerobic and strength training for at least 45 min per day, at least six days per week; and sleep health was supported by seven to eight hours of quality sleep per night; stress management included biofeedback and heart rate variability training; cognitive exercises were conducted for 15 min per day targeting speed and accuracy of information processing; and finally, hormonal and vitamin status was optimized along with anti-inflammatory dietary components to provide near-best lifestyle factors to improve cognitive health [187]. By three months continuing until nine months of treatment, twenty-one patients improved their CNS Vital Signs scores, Montreal Cognitive Assessment scores, Alzheimer’s questionnaire scores, and MRI volumetrics scores were also improved with a personalized medical protocol with modifiable lifestyle factors [187]. However, it is important to note the small sample size of this study and the limited generalizability of its findings due to potential confounding biases, such as participant motivation, and the need for replication of this study with larger cohorts from diverse populations using blinded controls to substantiate the reported results and claims.

In addition, the role of estrogen loss, neuroinflammation, and cognitive decline is presented in Section 4 above. However, there are additional key features of the role of estrogen in cognitive aging [52]. Other important factors associated with estrogen are as follows: (a) the systemic inflammatory phase during perimenopause that enables later neurodegeneration to occur, especially given that inflammasomes have been shown to be involved in mitochondrial dysfunction [145,188,189,190]; and (b) dysregulation of the immune system, which has been linked to a broad range of diseases, from diabetes to neurodegeneration, where the body–brain circuit monitors the development and progression of inflammatory responses and ensures or tries to bring it back into homeostasis between pro-inflammatory and anti-inflammatory states [190] (Figure 9). In this regard, recent CRISPR-based studies published in 2025 investigating sex-specific inflammasome knockouts in animal models have been reported [191]. The authors utilized a mouse model of monkeypox virus infection, using CRISPR knockout of the inflammasome sensor and associated components revealed their central role in driving inflammasome activation, pyroptotic cell death, and the release of pro-inflammatory cytokines such as IL-1β and IL-18 [191]. These findings used genetically altered mice to show how disrupting these nodes profoundly alters inflammatory responses to viral challenges and help clarify underlying mechanisms for therapeutic targets like NLRP3 inhibitors [192].

Due to the lack of therapeutic options for neurodegenerative diseases, recent attention has shifted toward addressing modifiable risk factors such as unhealthy diets and lifestyles to slow down the onset of AD, where neuroinflammation is a major contributor to neurodegeneration [193], which was covered in Section 4. In addition, to emphasize this point, the three major neuropathological characteristics in AD are as follows: first, the accumulation of amyloid-β; second, tubulin (tau) fibrillation in neurons; and third, activation of microglial and neuroinflammation in the brain [193], where this last topic represents the best option to treat neurodegeneration through improvements in lifestyle factors.

## 7. Current and Future Directions

Aging as a health topic has become an important area of science and medical technology aimed at slowing down aging, finding early detection, and hopefully treating age-related dysfunctions, disorders, and diseases [194]. This is evident as cardiovascular disease, cancer, metabolic disorders (like obesity and type 2 diabetes), and neurodegenerative diseases represent the four major cornerstones of age-related diseases [195]. A recent report from neuroscience investigators at the University of Cambridge, United Kingdom, identified four milestones at ages 9, 32, 66, and 83 that create five broad eras of neural wiring over the human lifespan, which may mirror how the human brain experiences development over time and warrants further investigation in relation with aging and neurodegeneration [196]. Aging, especially for women with hormonal changes associated with estrogen decline and loss during midlife and menopause, represents a transition that negatively impacts almost every tissue and organ in the body, including the brain, therefore causing neurodegeneration (Figure 3). However, with the re-evaluation of the WHI study results from 2002, the transformation of scientific and medical evidence demonstrating that HRT can provide protective health benefits, and the recent reversal by the US FDA to withdraw stringent warnings on hormone therapy products for menopausal women, saying that these treatments offer heart, brain, and bone health benefits [176], it can now be envisioned that HRT may become more commonplace in the future. Moreover, HRT, and especially estrogen replacement, appears to play a major role in this therapeutic approach that may delay the onset of, and potentially slow down the progression of, AD. From this perspective, round table discussions are becoming increasingly more common, bringing together specialists like neuroscientists, neurologists, psychiatrists, and gynecologists to explore innovative treatments and make recommendations for perimenopausal and menopausal patients [197], because an interdisciplinary approach is necessary to address women’s health, menopause, and AD.

In addition, lifestyle factors such as nutrition have come to the forefront for healthy living, where polyphenols, phytoestrogens, and carotenoids have been shown to help slow aging by acting as powerful antioxidants and anti-inflammatory compounds that neutralize harmful free radicals, thereby reducing cellular damage, inflammation, and the risk of age-related diseases, including AD [17,18,198]. Currently, there is no easy diagnostic measurement to determine whether an individual is consuming adequate fruits and vegetables without blood analysis, which is invasive, labor-intensive, and expensive. However, the MEDiet is one of the most widely studied diets and probably has among the highest carotenoid content due to its high proportion of fruits and vegetables, which appears to enhance health and potentially enhance lifespan [198]. It has been shown that non-invasive estimations of carotenoid food and/or supplement intake can be determined by skin carotenoid scans (SCSs), since these compounds are deposited in the skin, which may be a promising way to potentially assess carotenoid levels that could slow down the onset and/or progression of AD, since portable carotenoids skin scanners are becoming increasingly available [198,199]. Validation studies of skin carotenoid scans have a high correlation with carotenoid levels quantified in blood samples, and potential confounders have been reported [198]. For example, in 2025, skin carotenoid scores were reported from twenty different countries, representing thousands to millions of scans per country. The highest skin carotenoid scores were recorded in Korea, while the lowest scores were observed in Indonesia, which has one of the highest smoking rates in the world and may account for the low carotenoid levels recorded in this country [198]. The Philippines also displayed low skin carotenoid scores, which were accounted for by malnutrition, while obesity is also known to decrease carotenoid levels [198]. Therefore, skin carotenoid levels, which have been shown to correlate with dietary intake, may provide potential benefits for reducing AD risk; however, this warrants further study.

It is important to review the World Health Organization’s 2025 report on dementia, since this disorder is one of the most pressing public health challenges, affecting tens of millions of people worldwide and imposing significant social and economic burdens, where accelerated efforts are urgently needed to improve prevention, diagnosis, and care [200]. In addition, the report emphasizes the need to support people with dementia and to provide interventions that address barriers to care, particularly for vulnerable populations, especially women, since they represent the largest population with AD [200]. Notably, this presentation aligns with the WHO perspective and its proposed goals.

Thus, with aging in women, several interconnected factors contribute to changes in brain health, notably estrogen loss, diet, and neuroinflammation. The decline in estrogen during midlife and menopause removes a key neuroprotective influence, as estrogen normally supports synaptic plasticity, mitochondrial function, and regulation of inflammatory responses in the brain. This hormonal shift can increase vulnerability to neuroinflammation, a process that becomes more pronounced with age and is linked to cognitive decline and neurodegenerative risk. Diet further modulates these processes, either exacerbating or mitigating inflammation. Nutrient-poor, highly processed diets can promote systemic and central inflammation, while diets rich in antioxidants, healthy fats, and anti-inflammatory compounds help counteract inflammatory pathways and support neural resilience. Together, estrogen loss, dietary patterns, and age-related increases in neuroinflammation interact to shape brain aging in women, highlighting the importance of hormonal, nutritional, and inflammatory factors in maintaining cognitive health across the lifespan.

Table 2 outlines the proposed time intervals, causal interactions, and influential relationships among the four pillars covered in this narrative overview (see below).

## 8. Strengths and Limitations

This narrative overview provides a readable, thoughtful, and practical exploration of the critical combination of factors, such as aging, the decline and loss of estrogen in women during perimenopause and menopause, the important positive aspects of nutrition, and the mechanisms of neuroinflammation that takes place with these changes and can lead to AD. The review authors advance this new perspective by describing and interpreting literature in the field, along with their training and experience in these research areas, which required detailed and integrative interpretations of complex focal components while maintaining a broad perspective [201,202]. This narrative overview does not aim to be systematic syntheses that answer(s) specific, highly focused question(s); instead, it offers an expansive perspective joining together a body of research knowledge to propose novel strategies to delay the onset of and/or slow down the progression of AD, especially in women. This overview does not provide an exhaustive, critical/comprehensive review of the literature; however, the usefulness of this article is to stimulate new ideas and consider the use of HRT and lifestyle factors that may potentially decrease the incidence of neurodegeneration and dementia. Notably, this combination of topics will be featured in a future Special Issue in *Frontiers in Neuroscience* in 2026.

## Figures and Tables

**Table 1:** Comparison of Alzheimer’s disease characteristics with other dementias [1,2,3,7,10,13].

Feature	Alzheimer’s Disease	Other Dementias (Vascular, Lewy Body, and Frontal/ Temporal)
First symptoms	Memory loss	Varies (behavior, attention, and motor)
Progression	Gradual and steady	Stepwise or fluctuating
Age of onset	Usually > 65 years old (yo)	Earlier in frontal/temporal dementia (FTD) (45–65 yo)
Hallucination	Late	Early in Lewy body dementia (LBD)
Motor symptoms	Late	Early in (LBD)/Parkinson’s dementia
Pathology	Amyloid β and tau proteins	Vascular *, alpha-synuclein, and TDP-43 protein

* strokes and ischemic events; TDP-43 (transactive response DNA-binding protein 43 kDa).

**Table 2:** Relationships/interactions among the four pillars (aging, estrogen loss, diet/nutrition, and neuroinflammation).

AGING:	Alzheimer’s disease in women is closely linked to aging and reflects a combination of biological, hormonal, and social factors that influence risk and progression. As women age, they experience a higher lifetime risk of developing Alzheimer’s disease than men, partly because they tend to live longer, but also due to sex-specific changes in the brain and body. In the United States, a woman’s lifetime risk of developing Alzheimer’s disease at age 65 is one in six, compared to one in eleven for a man.
CHANGES IN ESTROGEN:	One important factor is the decline in estrogen during perimenopause, starting around ages 35–45, and menopause, which occurs between 46 and 55 years of age (with the average age at menopause around 51–52 years of age). Estrogen supports brain glucose metabolism, synaptic plasticity, mitochondrial function, and regulation of neuroinflammation, along with many other protective brain effects. Estrogen loss can increase vulnerability to neuroinflammation, a process that becomes more pronounced with age and is linked to cognitive decline and neurodegenerative risk.
DIET/NUTRITION:	Diet further modulates these processes, either exacerbating or mitigating inflammation. Nutrient-poor, highly processed diets can promote systemic and central inflammation, while diets rich in antioxidants, healthy fats, and anti-inflammatory compounds help counteract inflammatory pathways and support neural resilience.
NEUROINFLAMMATION:	Neuroinflammation arises from immune dysregulation, oxidative stress, impaired brain repair mechanisms, and the presence/increase in amyloid-beta plaques and hyperphosphorylated tau proteins that create neurofibrillary tangles. Aging primes the brain for chronic inflammation, while estrogen loss removes critical anti-inflammatory protection and weakens the blood–brain barrier. Diet can either worsen or mitigate inflammatory signaling, like polyphenolic compounds, omega-3 fatty acids, and fiber to support the gut–brain axis and gut microbiome.

## Data Availability

No new data were created or analyzed in this study. Data sharing is not applicable to this article.

## Abbreviations

The following abbreviations are used in this manuscript:

α	alpha
β	beta
γ	gamma
µg	microgram
A	androstenedione
AD	Alzheimer’s disease
DHEA	dehydroepiandrosterone
DHEAS	dehydroepiandrosterone sulfate
dL	deciliter
AC	adenylyl cyclase
AFT-4	activating transcription factor 4
Akt	a group of enzymes involved in important cell signaling pathways; often referred to as protein kinase B (PKB); a family of three serine/threonine proteins
AQP4	aquaporin 4
APE1	apurinic/apyrimidinic endonuclease 1
APOE4	apolipoprotein E4
Arg-1	arginase-1
AMPK	AMP-activated protein kinase
ARE	activation response element
ATP	adenosine triphosphate
BAX	pro-apoptotic protein
BDNF	brain-derived neurotrophic factor
BBB	blood–brain barrier
BCL-2	anti-apoptosis regulator
BCL-X	inhibits programmed cell death (xL), (xS) promotes programmed cell death
BPH	benign prostatic hyperplasia
cAMP	cyclic adenosine monophosphate
cGMP	cyclic guanosine monophosphate
C1q	immune response protein (complement)
CCLs	chemokines
CD206	mannose receptor C type 1, immune regulator
Chi313	chitinase 3-like 3
COX-3	cyclooxygenase 3
CREB	cAMP response element-binding protein
CRP	C-reactive protein
CtyC	cytochrome C
CSF	cerebral spinal fluid
CYP 19 A1	cytochrome P450 19 gene A1 (human aromatase)
CXCLs	chemokine (C-X-C motif), immune/inflammation signals
Da	Dalton (unit of molecular with in proteins)
DNA	deoxyribonucleic acid
eNOS	endothelial nitric oxide synthase
ER	estrogen receptor
ERE	estrogen-responsive element
ERK1/2	extracellular signal-regulated kinase 1/2
FIZZ1	inflammatory zone 1
FSH	follicle-stimulating hormone
FTD	frontal/temporal dementia
GFAP	glial fibrillary acidic protein
GLAST	glutamate aspartate transporter
GLT-1	glutamate transporter 1
GM-CSF	granulocyte–macrophage colony-stimulating factor
GPER	G-protein-coupled estrogen receptor
G proteins	(α, β, γ) subunits
GPx	glutathione peroxidase
GSH	glutathione
GSM	genitourinary syndrome of menopause
HMGB1	high-mobility group protein
HO-1	refers to the Keap1-Nrf2 pathway where HO-1 (heme oxygenase-1) is a key downstream target
HRT	hormone replacement therapy
HSP	heat shock protein
IFN-β	cytokine produced by microglial
IFN-γ	interferon gamma
IGF-1	insulin-like growth factor 1
L	liter
IL-1β	interleukin 1 beta
IL-4	interleukin 4
IL-6	interleukin 6
IL-8	interleukin 8
IL-10	interleukin 10
IL-12	interleukin 12
IL-13	interleukin 13
IL-23	interleukin 23
IP3	inositol triphosphate
UL	international units
LBD	Lewy body dementia
LPS	lipopolysaccharide
LUTS	lower urinary tract symptoms
MEDiet	Mediterranean diet
mg	milligram
MHC-I	major histocompatibility complex I
MHC-II	major histocompatibility complex II
MHT	menopause hormone therapy
ml	milliliter
MAPK	mitogen-activated protein kinase
MEK	mitogen-activated protein kinase
Mn-SOD	mitochondrial manganese superoxide dismutase (known as SOD 2)
MRI	magnetic resonance imaging
mTOR	mammalian target of rapamycin
NAMS	North American Menopause Society
NFk-B	nuclear factor kappa-light-chain-enhancer of activated B cells; pro-inflammatory signaling pathway
ng	nanogram
NLRP3	NLR family pyrin domain-containing 3
NO	nitric oxide
NQO-1	NAD(P)H quinone dehydrogenase 1
NRF-1	nuclear respiratory factor 1
Nrf2	nuclear factor erythroid 2-related factor 2
OH-1	heme oxygenase-1
PGC-1α	peroxisome proliferator-activated receptor gamma coactivator 1-alpha
PI3K	phosphatidylinositol 3-kinase
PKB	protein kinase B
PLC	phospholipase C
RAS/RAF	cell signal transduction pathway
ROS	reactive oxygen species
SCR	Scr family kinases
SERM	selective estrogen receptor modulator
SIRT1	NAD-dependent deacetylase surtuin-1
SOC3	suppressor of cytokine signaling 3
SOD	superoxide dismutase
STAT1	signal transducer and activator of transcription 1, immune response
STAT3	signal transducer and activator of transcription 3, immune response
STAT6	signal transducer and activator of transcription 6, immune response
T	testosterone
TDP-43	transactive response DNA-binding protein 43 kDa
TF	transcription factors
TFAM	mitochondrial transcription factor A
TLR4	Toll-like receptor 4, immune response
TNF-α	tumor necrosis factor alpha
USA.	United States of America
US FDA	United States Food and Drug Administration
USD	United States Dollar
Ym1	secreted by activated macrophages during inflammation
